# Metabolome Analysis Revealed the Mechanism of Exogenous Glutathione to Alleviate Cadmium Stress in Maize (*Zea mays* L.) Seedlings

**DOI:** 10.3390/plants10010105

**Published:** 2021-01-06

**Authors:** Runfeng Wang, Kaina Lin, Huabin Chen, Zhenyu Qi, Bohan Liu, Fangbin Cao, Hao Chen, Feibo Wu

**Affiliations:** 1 Institute of Crop Science, College of Agriculture and Biotechnology, Zhejiang University, Yu-Hang-Tang Road No 866, Hangzhou 310058, China; 11516025@zju.edu.cn (R.W.); linkaina@zju.edu.cn (K.L.); 21816033@zju.edu.cn (H.C.); liubohan@zju.edu.cn (B.L.); wufeibo@zju.edu.cn (F.W.); 2 Experimental Station, Zhejiang University, Yu-Hang-Tang Road No 866, Hangzhou 310058, China; qizhenyu@zju.edu.cn; 3 Southern Regional Collaborative Innovation Center for Grain and Oil Crops in China, College of Agriculture, Hunan Agricultural University, Changsha 410128, China

**Keywords:** cadmium stress, glutathione, metabolome analysis, maize

## Abstract

Cadmium (Cd) is one of the major heavy metal pollutants in the environment and imposes severe limitations on crop growth and production. Glutathione (GSH) plays an important role in plant Cd tolerance which is able to scavenge stresses-induced reactive oxygen species (ROS) and is involved in the biosynthesis of phytochelatins (PCs). Our previous study revealed that Cd stress affects maize growth, and the GSH treatment could relieve Cd stress in maize seedlings. In this study, we attempted to characterize the metabolomics changes in maize leaves and roots under Cd stress and exogenous GSH conditions. We identified 145 and 133 metabolites in the leaves and roots, respectively. Cd stress decreased the tricarboxylic acid cycle (TCA cycle) metabolism and increased the amino acid contents in the leaves, while it decreased the amino acid contents, increased the TCA cycle metabolism, the sugar contents, and shikimic acid metabolism in the roots. On the other hand, exogenous GSH increased the GSH content, changed the production of metabolites related to antioxidant systems (such as ascorbic acid-related metabolites and flavonoid-related metabolites), and alleviated lipid peroxidation, thereby alleviating the toxic effect of Cd stress on maize. These findings support the idea that GSH alleviates Cd-induced stress in maize and may help to elucidate the mechanism governing Cd-induced stress and the GSH-driven alleviation effect.

## 1. Introduction

Cd has become one of the major heavy metal pollutants in the environment. Generally, the concentration of Cd is very low in the natural environment. However, various human activities, such as zinc mining and smelting and the use of fertilizers, pesticides, and fungicides, lead to increased Cd concentrations in the soil [1,2,3]. Due to the high mobility of Cd, it can be absorbed easily by plants and accumulated and transferred through the food chain, thus threatening human health. As a result that Cd is a non-essential element in the human body, excessive exposure may lead to cancer, bone damage, lung dysfunction, and other symptoms [4]. The absorption and accumulation of Cd in plants cause a series of morphological, physiological, and biochemical changes, and it can also affect many metabolic processes. Therefore, it is necessary to develop strategies to reduce Cd accumulation in maize for safe food production.

Metabolomics can systematically explore the distribution of metabolites in organisms, help to identify and quantify the metabolites involved in the primary metabolic pathway, such as sugars, alcohols, amino acids, organic acids, and polyamines, and provide valuable information about organismal responses to internal and external interference [5]. A proteomic and metabolomic study on Arabidopsis showed that the primary response of the metabolome to Cd stress was to activate the carbon, nitrogen, and sulfur metabolism, thus triggering the synthesis of Cd-chelating molecules (phytochelatins, etc.) [6]. Xie et al. [7] analyzed the differences in the metabolites between Cd-resistant and Cd sensitive-genotypes in Bermuda grass by Gas Chromatography-Mass Spectrometer (GC-MS), and they found that differences in Cd tolerance could be attributed to different accumulation levels of amino acids (proline, aspartic acid, alanine, glycine, asparagine, and serine), organic acids (citric acid, malic acid, and oxalic acid), and sugars (trehalose, fructose, and galactose). Cd stress affects plant photosynthesis, and oxidative stress seriously affects plant primary metabolism, which causes plants to obtain more energy from other metabolic pathways (such as glycolysis, the TCA cycle, etc.) to maintain plant growth and resist the toxic effects induced by stress [6,8,9]. Therefore, it is necessary to elucidate the physiological mechanism of maize in response to Cd stress at the metabolic level.

Glutathione (GSH) is a major nonprotein tripeptide thiol in plant cells, and it is involved in cell differentiation, free radical and hydrogen peroxide scavenging, thiol-disulfide exchange, and phytochelatin synthesis [10,11,12,13,14]. Studies on heavy metal tolerance or metal hyperaccumulators have shown that the GSH biosynthesis and regeneration enzyme activities had significant effects on the heavy metal tolerance of plants [15]. Iannelli et al. [16] showed that high GSH and ASA contents and the APX, CAT, GR, GST, and GPX activities were the key reasons for Cd tolerance in *Phragmites australis*. In addition, GSH-mediated ROS and Mg metabolism were also reportedly involved in plant heavy metal tolerance [13,17,18,19,20].

In recent years, there have been many reports on the mitigation of the toxic effects of Cd stress in plants by GSH, but there have been few metabolomics analyses in maize [10,13,18,19]. Our previous study has been found that exogenous GSH significantly alleviated Cd-induced growth inhibition. Hence, in this study, the metabolites of maize leaves and roots were analyzed by GC-MS to understand the physiological mechanism of exogenous glutathione to alleviate cadmium stress at the metabolic level in the maize seedlings.

## 2. Results

### 2.1. Metabolite Analysis

**Figure 1:**
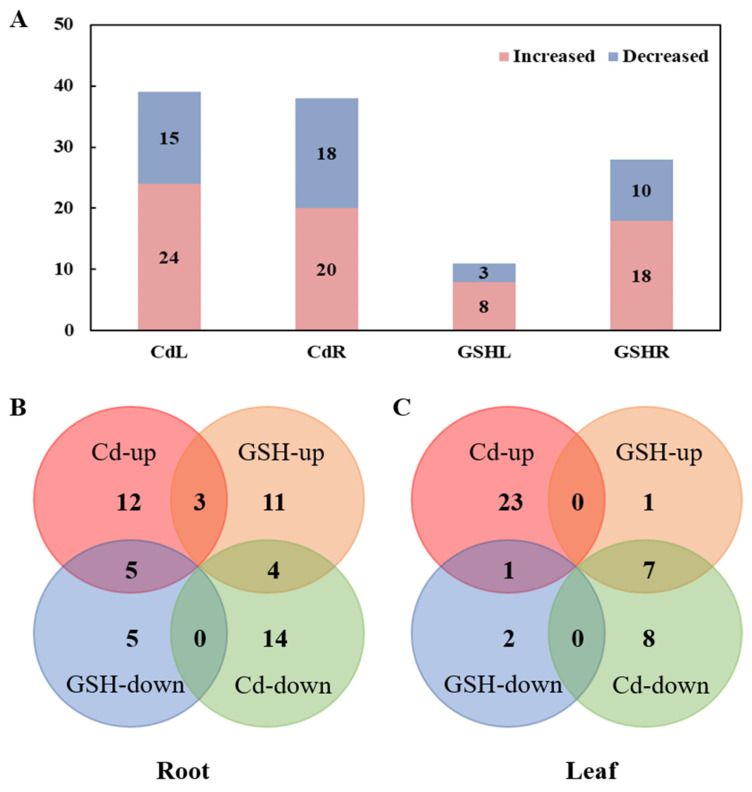
The number of different metabolites in the roots and leaves of maize seedlings under exogenous glutathione (GSH) and cadmium stress conditions. (**A**) Increased and decreased metabolites in the roots and leaves of maize seedlings under exogenous GSH and cadmium stress conditions. (**B**) Venn diagram of increased and decreased metabolites in the roots of maize under exogenous GSH and cadmium stress conditions. (**C**) Venn diagram of increased and decreased metabolites in maize leaves under exogenous GSH and cadmium stress conditions. CdL, Cd treatment vs. control in leaves; CdR, Cd treatment vs. control in roots; GSHL, exogenous GSH and Cd treatment vs. Cd treatment in leaves; and GSHR, exogenous GSH and Cd treatment vs. Cd treatment in roots. Cd treatment (5 μM CdCl_2_), exogenous GSH (30 μM GSH).

**Figure 2:**
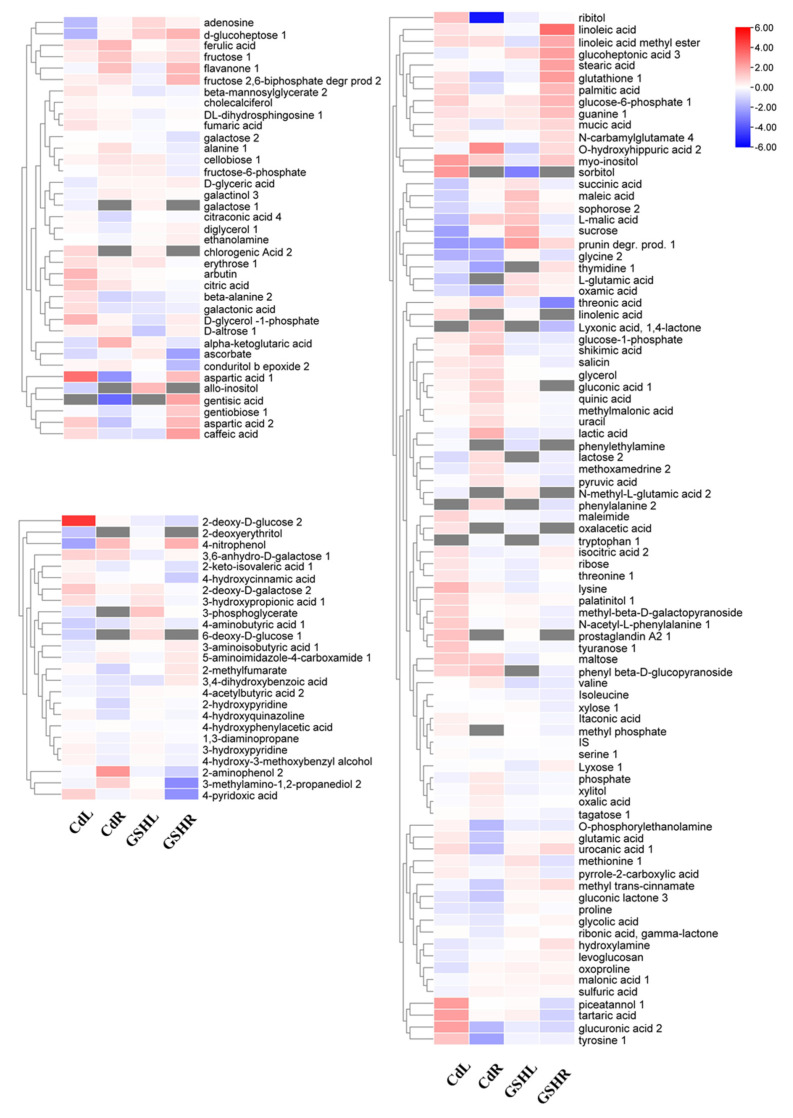
Heat map analysis combined with hierarchical cluster analysis of the metabolomics in maize roots and leaves under exogenous GSH and cadmium stress conditions. The changes in the metabolites of maize roots and leaves under exogenous GSH and cadmium stress conditions are shown in the heat map. Cd treatment (5 μM CdCl_2_)., exogenous GSH (30 μM GSH).

Totally, 150 metabolites were identified by GC-MS. There are 145 and 133 metabolites in the leaf and root tissues, respectively. To show the effects of Cd stress and exogenous GSH on the metabolomics of the maize leaves and roots, we calculated the fold change (FC) in the metabolites from the maize leaves and roots under Cd stress and exogenous GSH conditions. In roots, compared with the control condition, the contents of 20 metabolites increased and 18 metabolites decreased under Cd treatment, involving glutamic acid, glutathione, maltose, shikimic acid; 18 metabolites increased and 10 metabolites decreased after exogenous GSH addition (Figure 1A). Among these metabolites, 5 metabolites increased under Cd stress but decreased after exogenous GSH addition, 3 metabolites that increased under Cd stress and exogenous GSH addition, and 4 metabolites that decreased under Cd stress but increased after exogenous GSH addition (Figure 1B). In the leaves, 24 metabolites increased and 15 metabolites decreased under Cd treatment, and 8 metabolites increased and 3 decreased after exogenous GSH addition (Figure 1A). Among them, only 1 metabolite in leaves increased under Cd stress but decreased after exogenous GSH addition, and 7 metabolites decreased under Cd stress but increased after exogenous GSH was applied (Figure 1C). The fold changes of all the metabolites are represented by heatmap diagrams (Figure 2). The results of the cluster analysis showed that the metabolite variation trend was the largest among the leaves and roots, and the variation trend in metabolites between Cd stress and GSH treatment was also quite different.

### 2.2. Leaf and Root Metabolite Response to Cd Stress

**Figure 3:**
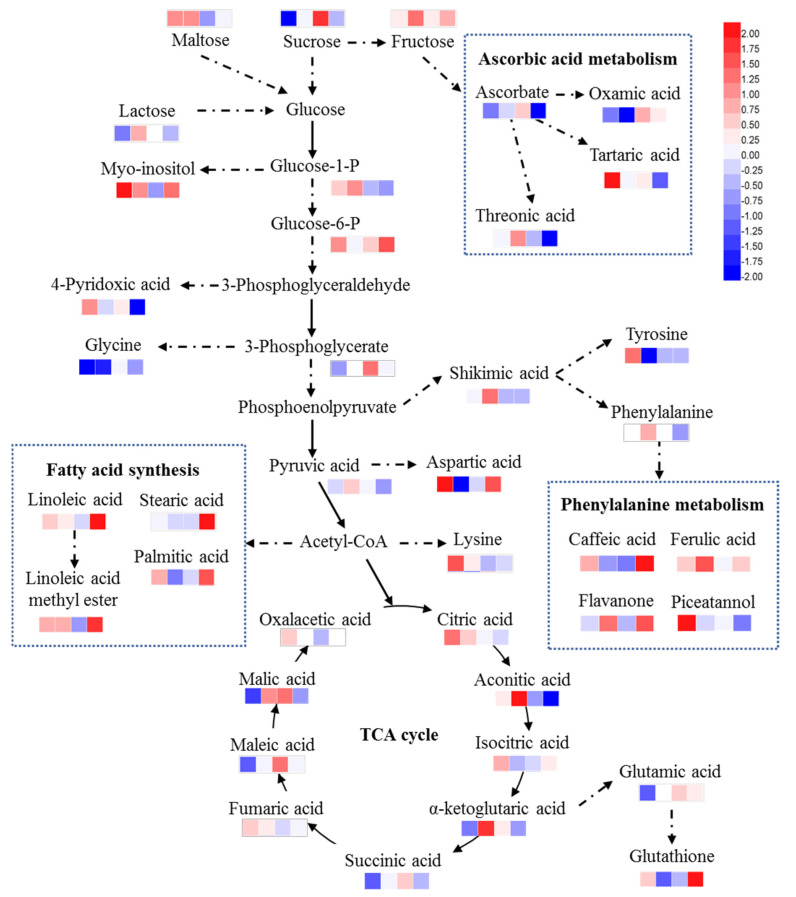
Changes in major metabolic pathways of maize roots and leaves under exogenous GSH and cadmium stress conditions. The solid arrows represent direct metabolic pathway connections, and the dashed arrows indicate that these metabolites are involved in the metabolic pathway. The changes in the metabolites in maize roots and leaves under exogenous GSH and cadmium stress conditions are shown in the heatmap, and their order within the heatmap is CdL, CdR, GSHL, and GSHR. CdL, Cd treatment vs. control in leaves; CdR, Cd treatment vs. control in roots; GSHL, exogenous GSH and Cd treatment vs. Cd treatment in leaves; and GSHR, exogenous GSH and Cd treatment vs. Cd treatment in roots. Cd treatment (5 μM CdCl_2_), exogenous GSH (30 μM GSH).

The heatmap results showed that there were significant differences in the root and leaf metabolite responses to Cd stress. Compared with the control, 39 and 37 metabolites were changed under Cd stress in roots and leaves, respectively, and only 4 metabolites (cherry glycoside degradation product 1, glycine, maltose, and inositol) displayed the same trend in both roots and leaves. In the leaves, 15 metabolites decreased significantly, including sugar and their related metabolites, such as sucrose, D-glucoheptose, sophorose, 2-deoxyerythritol, and 6-deoxy-D-glucose; TCA cycle-related metabolites, such L-malic acid, succinic acid, and maleic acid; and other metabolites including glycine, glutamine acid, 4-aminobutyric acid, and iso-inositol (Table 1, Table S1, Figure 1A and Figure 3). Twenty-four metabolites were significantly increased, including amino acids and their related metabolites, such as aspartic acid, tyrosine, lysine, and N-acetyl-L-phenylalanine; sugars and their related metabolites, such as maltose, 2-anhydro-D-galactose, methyl-β-D-galactopyranoside, palatinitol, glucose-6-phosphate, 2-deoxy-d-galactose, 2-deoxy-D-glucose, turanose, glucuronic acid, myo-inositol, and sorbitol; and other metabolites including 4-pyridoxic acid, citric acid, arbutin, tartaric acid, piceatannol, and 1-glycerophosphate (Table 1, Table S1 and Figure 1A). These results suggested that Cd stress decreased the TCA cycle metabolism and increased the amino acid contents in leaves.

In the roots, 17 metabolites decreased significantly under Cd stress, including amino acids and their related metabolites, such as aspartic acid, glutamic acid, glycine, tyrosine, and β-alanine; and other metabolites including glutathione, 2-methyl fumarate, thymidine, and glucuronic acid (Table 1, Table S1 and Figure 1A). Twenty metabolites in the roots of the maize seedlings increased under Cd stress, including sugars and their related metabolites, such as fructose, maltose, gluconic acid, glucose-1-phosphate, phenyl beta-D-glucopyranoside, threonic acid, and lyxonic acid-1,4-lactone; TCA cycle-related metabolites such as α-ketoglutaric acid, malic acid, and aconitic acid; shikimic acid metabolism-related metabolites such as shikimic acid, flavanone, ferulic acid, and quinic acid, and other metabolites including myo-inositol, lactic acid, and hydroxyhippuric acid (Table 1, Table S1 and Figure 1A). These results suggested that Cd stress decreased the amino acid contents, increased the sugar contents, and increased the TCA cycle metabolism and shikimic acid metabolism in the maize roots.

### 2.3. Leaf and Root Metabolite Responses to Exogenous GSH Addition

Twenty-eight and 11 differential metabolites were identified after exogenous GSH addition in the maize roots and leaves, respectively, compared with the Cd treatment (Figure 1A), among which only D-glucoheptanose showed a significant increase in both the roots and leaves. In the leaves, the sorbitol, altrose, and hydroxyhippuric acid contents decreased significantly under exogenous GSH compared with the Cd treatment, while the sucrose, d-heptanose, sophorose, 3-phosphoglycerate, malic acid, maleic acid, allo-inositol, and prunin degr. prod. 1 content increased significantly (Table 1, Table S1). Ten metabolites decreased in the roots, including aconitic acid, threonic acid, 4-pyridoxic acid, ascorbate, lyxonic acid-1,4-lactone, 4-hydroxycinnamic acid, and tartaric acid, while 18 metabolites increased, including sugars and their related metabolites such as fructose-2,6-diphosphate, glucose-6-phosphate, D-glucoheptose, gentiobiose, glucoheptanic acid, and inositol; fatty acid-related metabolites such as stearic acid, palmitic acid, linoleic acid, and linoleic acid methyl ester as well as guanine, aspartic acid, flavanone, caffeic acid, and glutathione (Table 1, Table S1). Combined with the concentration changes in these different metabolites under Cd stress, most of the metabolites showed the opposite change trend after exogenous GSH addition, which could reflect the mitigation effect of exogenous GSH on Cd stress (Figure 1, Table S1).

## 3. Discussion

Our previous study has found that exogenous GSH could significantly alleviate the toxic effect of Cd stress in maize [21]. However, the mechanism of the alleviating effects of GSH on Cd stress has been largely unknown at the metabolic level. Our study analyzed the metabolites responses to exogenous GSH under Cd stress. Based on the differential accumulate metabolites, we proposed a working model to explain how GSH mitigates Cd toxicity in maize seedlings (Figure 3).

### 3.1. GSH Alleviated the Inhibition of Cd Stress on TCA Cycle and Sugar Metabolism in Maize

Oxidative stress induced by Cd may directly change the TCA cycle-related metabolites. It was reported that enzymes containing [Fe-S] clusters, such as aconitase (E.C.4.2.1.3, with aconitic acid or citric acid as substrates, isocitric acid or aconitic acid as products), fumarate hydratase (E.C.4.2.1.2, malic acid as substrates and fumaric acid as products), and enzymes containing thiol groups were particularly sensitive to reactive oxygen species [22,23]. The accumulation of substrates caused by the inactivation of ROS-modified enzymes may also be the reason for several increased metabolites in the TCA cycle. We also found that the malic acid and maleic acid in the leaves increased and the aconitic acid in the roots decreased after exogenous GSH addition, contrary to their trend under Cd stress (Table 1, Figure 3), indicating that exogenous GSH may alleviate the inhibition of Cd stress on the TCA cycle by alleviating oxidative stress.

Cd stress affects plant growth and metabolism, including amino acid and sugar metabolism. Shahid et al. [24] observed that Cd treatment significantly increased the concentrations of sucrose, fructose, and glucose in potatoes, and these sugars increased more under Cd and selenium treatment. The accumulation of soluble sugars in plants helps to maintain the water status under stress and reduces the adverse effects of ROS [25]. Our study also found that Cd stress increased the maltose contents in the leaves and increased the fructose and maltose contents in the roots (Table 1, Table S1 and Figure 3). Compared with Cd stress, exogenous GSH addition increased the contents of heptanose, sophorose, and sucrose in the maize leaves, and these sugars decreased under Cd stress (Table 1, Figure 3), indicating that exogenous GSH alleviated the inhibition of Cd stress on sugar metabolism in maize.

### 3.2. GSH Alleviated the Plant Response to Cd Stress with Osmotic Metabolites

Several studies have suggested that some metabolites that accumulate under stress can be used as osmotic protectants to stabilize proteins and cell membranes, such as sugars, amines, amino acids, and polyols [26,27,28]. Sun et al. [29] found that the alanine, proline, serine, putrescine, sucrose, 4-aminobutyric acid, inositol, and other metabolite contents in *Arabidopsis thaliana* were increased under Cd stress. The accumulation of osmotic protectants (also called compatible solutes) is considered a common defense mechanism for plants to cope with stress. These osmotic metabolites have high solubility in cells and do not inhibit enzyme activity at high concentrations, which have certain protective effects on proteins and cell membranes [5]. Our study indicated that maltose, inositol, sorbitol, palatinitol, aspartic acid, lysine, and tyrosine increased in the leaves under Cd stress (Table 1, Figure 3). Compared with Cd stress, exogenous GSH increased the allo-inositol content and decreased the sorbitol content in the leaves, while allo-inositol decreased and sorbitol increased under Cd stress, indicating that GSH alleviated the plant response to Cd stress with osmotic metabolites.

### 3.3. Exogenous GSH Increased the Contents of GSH in Roots

As an important component of the plant antioxidant system, glutathione (GSH) plays a central role in maintaining cellular redox homeostasis. GSH is also a precursor of phytochelatins, which play a key role in the chelation and tolerance of heavy metals. A large number of studies have demonstrated the role of GSH in regulating tolerance to heavy metal or metalloid stress in plants [17,30]. In this study, we found that compared with Cd stress, exogenous GSH increased the contents of GSH in roots, which may be of great help to relieve Cd stress.

### 3.4. Exogenous GSH Increased the Contents of Flavonoids Related Metabolites

Flavonoids have strong antioxidant activity, which can help plants to eliminate reactive oxygen species [31]. Studies have shown that low Cd concentration induced the production of phenols and flavonoids in plants [32]. Sun et al. [29] found that under Cd stress, there was increased erucic acid, an intermediate in the phenylalanine pathway, and the concentration of isoflavones also increased significantly. Our results indicated that some metabolites in the phenylalanine metabolism pathway increased under Cd stress, in that paclitaxel increased in the leaves and shikimic acid, ferulic acid, and flavanone increased in the roots (Table 1, Figure 3). Plants can resist Cd stress by stimulating metabolic processes involved in the antioxidant system, such as the synthesis of a large number of metabolites with antioxidant effects such as flavanone and caffeic acid [33]. The caffeic acid and flavanone contents increased after GSH addition (Table 1, Figure 3), which suggested that GSH promoted the plant response to Cd stress may be through the phenylalanine metabolism pathway.

### 3.5. Exogenous GSH Decreased the Contents of Ascorbate Related Metabolites

Ascorbic acid is also an important part of the plant’s antioxidant system. In this study, we found that ascorbic acid-related metabolites changed after Cd stress, the tartaric acid content increased in the leaves, the threonic acid content increased in the roots, and the oxamic acid content decreased in the roots (Table 1, Figure 3). Moreover, the contents of ascorbic acid-related metabolites in the roots decreased under exogenous GSH, such as the contents of ascorbate, threonic acid, and tartaric acid (Table 1, Figure 3), demonstrating that exogenous GSH may increase the consumption of ascorbic acid, thus alleviated Cd-induced oxidative stress.

### 3.6. Exogenous GSH Increased the Contents of Fatty Acids

It is somewhat surprising that fatty acid-related metabolites, such as linoleic acid, methyl linoleic acid, palmitic acid, and stearic acid, were significantly increased under exogenous GSH compared with Cd stress (Table 1, Figure 3), while there was no significant change in the lipid metabolism under Cd stress compared with the control. ROS induced by Cd stress cause lipid peroxidation in plants, changes the lipid environment, affects the membrane lipid fluidity and membrane protein activity, affects the membrane absorption of nutrients, and then affects plant growth and development [23,34]. Cd toxicity also affects lipid biochemistry by inhibiting biosynthetic pathways, leading to the reduction of unsaturated fatty acids [35]. Navarro et al. [36] found that the glyceride-related metabolites (including glycerophospholipids) decreased in rice under Cd stress, and they believed that this decrease was related to the decreasing plant growth rate or photosynthetic activity. These studies led to the conjecture that exogenous GSH may alleviate lipid peroxidation induced by Cd stress and promote lipid synthesis, including that of saturated and unsaturated fatty acids. The reasons for the increase in these fatty acids and their roles in Cd stress remain to be further explored.

In conclusion, exogenous GSH increased endogenous GSH content and the consumption of ascorbic acid, promoted the synthesis of fatty acids and flavonoids related metabolites in the roots, and reduced the oxidative stress, thus alleviated the effects in sugar metabolism, TCA cycle, and other metabolic pathways caused by Cd stress. These findings revealed the mechanism of exogenous GSH to alleviate Cd stress in maize seedlings at the metabolic level and may provide a theoretical basis for reducing Cd stress in the production of maize and other crops.

## 4. Materials and Methods

### 4.1. Plant Materials and Plant Growth

Maize seeds (Nongda108) were soaked in 2% H_2_O_2_ for 30 min, washed with distilled water three times, and then soaked in deionized water overnight. The seeds were germinated on germination paper according to our previous study [21]. These seeds were then put in the plant growth chamber (% °C/30 °C day/night temperature, 12 h/12 h light/dark). Five-day-old and uniform seedlings were selected and transplanted into a 5 L black plastic container containing 4.5 L of basic nutrient solution. The basic nutrient solution consisted of (in gL^−1^) KNO_3_, 101; Ca(NO_3_)_2_·4H_2_O, 236; MgSO_4_·7H_2_O, 246; NH_4_H_2_PO_4_ 115; H_3_BO_3_, 1.85; MnCl_2_·4H_2_O, 0.99; (NH_4_)_6_Mo_7_O_24_·4H_2_O, 12.36; ZnSO_4_·7H_2_O, 1.15; CuSO_4_·5H_2_O, 0.5; and Fe(III)-EDTA, 84.2. The pH of the nutrient solution was adjusted to 5.8 ± 0.1 with 1 M NaOH or 1 M HCl. The solution was continuously ventilated by air pumps and renewed every 2 days. 6 days after transplanting, these seedlings were divided into 3 groups with different treatments, each treatment contained 3 replicates: control (basal nutrient solution, BNS), Cd (BNS +5 μM CdCl_2_), and Cd + GSH (BNS + 5 μM CdCl_2_ + 30 μM GSH). After 5 days of treatment, the leaves and roots were collected and immediately frozen in liquid nitrogen and stored in a freezer at −80 °C for further analysis.

### 4.2. Metabolite Extraction

One hundred milligrams of each frozen sample was transferred into 2 mL EP tubes, and 400 μL of extraction solution (methanol/water = 3:1, *V/V*) and 20 μL of ribosol (2 mg/mL) were added to the tube and the mixtures were vortexed for 30 s. A steel ball was added to the sample tube and the tissue was ground for 4 min at 40 Hz with a grinder, followed by ultrasonic treatment for 5 min (ice water bath). Next, the sample tubes were centrifuged at 4 °C and 12,000 rpm for 15 min, and 350 μL of supernatant was collected and dispensed into a 2 mL injection bottle.

### 4.3. Metabolite Derivatization

The extracts were dried in a vacuum concentrator, 60 μL of methoxyamination hydrochloride (20 mg/mL dissolved in pyridine) was added, and the samples were mixed gently and incubated at 80 °C for 30 min. An 80 μL volume of BSTFA (1% TCMs, *V/V*) was added to the sample tube and each sample was mixed gently and incubated at 70 °C for 120 min.

### 4.4. GC-MS Analysis

GC-MS analysis was performed with an Agilent 7890 gas chromatograph system and Pegasus HT time-of-flight mass spectrometer. The system included a DB-5MS capillary column coated with 5% diphenyl cross-linked with 95% dimethylpolysiloxane (30 m 250 μm inner diameter, 0.25 μm film thickness; J&W Scientific, Folsom, CA, USA). One microliter of the test sample was injected in splitless mode. Helium was used as the carrier gas, the purge flow rate of the front inlet was 3 mL min^−1^, and the gas flow rate of the column was 1 mL min^−1^. The initial temperature was maintained at 50 °C for 1 min, increased to 300 °C at a rate of 10 °C min^−1^, and then maintained at 300 °C for 8 min. The front inlet, transmission line, and ion source temperatures were 280 °C, 270 °C, and 220 °C, respectively. The energy at electron impact mode was −70 ev. The mass spectrometry data were obtained in full-scan mode using a *m/z* range of 50–500 at a scanning rate of 20 spectra/sec after a solvent delay of 460 s. The stability of the system was measured by tracking the retention time (R.T. (in minutes)) of ribo-alcohol (internal standard).

### 4.5. Data Analysis

Chroma TOF4.3X software by LECO Corporation and a LECO-Fiehn Rtx5 database was used to extract the raw peaks, filter and calibrate the baseline data, align the peaks, analyze the deconvolution, identify the peaks, and integrate the peak area. The retention time index (RI) was used for peak identification, and the RI tolerance was 5000. The difference between the metabolites in each group was calculated by finding the ratio of the mean value and transforming it with log_2_. We set 1 as the threshold, log_2_FC ≥ 1 as the content increased, −1 < log_2_FC < 1 as the content was unchanged, and log_2_FC ≤ −1 for the decrease. The heatmap was drawn with HemI software (version 1.0, http://hemi.biocuckoo.org/) [37]. The metabolic pathway map was drawn with reference to the KEGG metabolic database (http://www.genome.jp/kegg/).

## Figures and Tables

**Table 1:** Different metabolites of major metabolic pathways in maize leaves and roots under cadmium treatment and exogenous GSH conditions.

Heading	Metabolites	FC (CdL)	FC (CdR)	FC (GSHL)	FC (GSHR)
Sugars	Fructose 1	0.41	1.34	0.39	0.86
	Gentiobiose 1	−0.09	−0.75	−0.15	1.27
	D-Glucoheptose 1	−1.74	0.23	1.16	1.75
	Maltose	1.24	1.02	−0.55	0.05
	Sophorose 2	−1.00	−0.23	1.20	0.26
	Sucrose	−2.23	0.20	1.84	−0.28
	Turanose 1	1.31	−0.03	−0.24	−0.22
Alcohols	Myo-inositol	2.37	1.19	−0.53	1.27
	Sorbitol	2.38	-	−3.01	-
	Allo-inositol	−1.04	-	1.64	-
	Palatinitol 1	1.07	0.12	0.29	0.12
Amino acids	Aspartic acid 1	3.38	−2.54	−0.13	1.56
	Aspartic acid 2	1.21	−1.38	−0.16	1.63
	β-Alanine 2	0.74	−1.03	−0.66	−0.23
	Glutamic acid	0.48	−1.31	0.16	0.19
	Glycine 2	−1.85	−1.56	0.21	−0.70
	L-Glutamic acid	−1.21	-	0.73	0.34
	Lysine	1.67	0.40	−0.46	−0.12
	Oxamic acid	−0.81	−1.91	0.81	0.25
	Tyrosine 1	1.40	−2.20	−0.28	−0.36
Glycolysis	3-Phosphoglycerate	−0.61	-	1.35	0.06
	Fructose-6-phosphate	−0.04	0.40	0.38	−0.41
	D-(glycerol 1-phosphate)	1.74	0.22	−0.72	0.47
	Fructose 2,6-biphosphate degr. prod	0.34	0.63	−0.27	1.71
	Glucose-1-phosphate	0.51	1.02	−0.44	−0.54
	Glucose-6-phosphate 1	1.13	0.22	0.66	1.72
TCA cycle	α-Ketoglutaric acid	−0.78	1.75	0.28	−0.61
	Citric acid	1.34	0.59	0.10	−0.15
	L-Malic acid	−1.49	1.14	1.35	−0.52
	Succinic acid	−1.24	0.22	0.65	−0.39
	Aconitic acid	0.31	3.69	−0.61	−3.30
	Maleic acid	−1.06	0.16	1.46	0.09
Phenylalanine metabolism	Shikimic acid	0.24	1.32	−0.43	−0.26
	Caffeic acid	0.86	−0.64	−0.76	2.27
	Ferulic acid	0.66	1.68	0.05	0.60
	Piceatannol 1	2.32	−0.01	0.08	−0.86
	Flavanone 1	−0.19	1.49	−0.40	1.66
Fatty acids	Linoleic acid	0.74	0.27	−0.15	3.42
	Linoleic acid methyl ester	0.90	0.84	−0.74	1.99
Fatty acids	Palmitic acid	0.90	−0.77	−0.02	1.65
	Stearic acid	0.12	−0.09	−0.23	2.23
Ascorbate metabolism	Ascorbate	−0.89	−0.24	0.54	−2.22
	Tartaric acid	2.28	0.14	0.33	−1.07
	Threonic acid	0.21	1.00	−0.41	−2.89

FC: Fold changes (Cd vs. control; GSH vs. Cd) for log_2_N. log_2_N ≥ 1 are increased, 0 < |log_2_N| < 1 are unchanged and log_2_N ≤ −1 are decreased. CdL, Cd treatment vs. control in leaves; CdR, Cd treatment vs. control in the roots; GSHL, exogenous GSH and Cd treatment vs. Cd treatment in leaves; and GSHR, exogenous GSH and Cd treatment vs. Cd treatment in roots. Cd treatment (5 μM CdCl_2_), exogenous GSH (30 μM GSH).

## Data Availability

The available data are contained within the article.

## Supplementary Materials

The following are available online at https://www.mdpi.com/2223-7747/10/1/105/s1. Table S1. Different metabolites in the leaves and roots of maize seedlings.

## References

[1] McBride M.B. (2003). Toxic metals in sewage sludge-amended soils: Has promotion of beneficial use discounted the risks?. Adv. Environ. Res..

[2] Li J., Xie Z.M., Xu J.M., Sun Y.F. (2006). Risk assessment for safety of soils and vegetables around a lead/zinc mine. Environ. Geochem. Health.

[3] Rao R.A.K., Kashifuddin M. (2016). Adsorption studies of Cd(II) on ball clay: Comparison with other natural clays. Arab. J. Chem..

[4] Shahid M., Dumat C., Khalid S., Niazi N.K., Antunes P.M.C. (2017). Cadmium Bioavailability, Uptake, Toxicity and Detoxification in Soil-Plant System. Rev. Environ. Contam. Toxicol..

[5] Ghatak A., Chaturvedi P., Weckwerth W. (2018). Metabolomics in Plant Stress Physiology. Adv. Biochem. Eng. Biol..

[6] Sarry J.E., Kuhn L., Ducruix C., Lafaye A., Junot C., Hugouvieux V., Jourdain A., Bastien O., Fievet J.B., Vailhen D. (2006). The early responses of Arabidopsis thaliana cells to cadmium exposure explored by protein and metabolite profiling analyses. Proteomics.

[7] Xie Y., Hu L.X., Du Z.M., Sun X.Y., Amombo E., Fan J.B., Fu J.M. (2014). Effects of Cadmium Exposure on Growth and Metabolic Profile of Bermudagrass [*Cynodon dactylon* (L.) Pers.]. PLoS ONE.

[8] Durand T.C., Sergeant K., Planchon S., Carpin S., Label P., Morabito D., Hausman J.F., Renaut J. (2010). Acute metal stress in Populus tremula x P. alba (717-1B4 genotype): Leaf and cambial proteome changes induced by cadmium(2+). Proteomics.

[9] Villiers F., Ducruix C., Hugouvieux V., Jarno N., Ezan E., Garin J., Junot C., Bourguignon J. (2011). Investigating the plant response to cadmium exposure by proteomic and metabolomic approaches. Proteomics.

[10] Waśkiewicz A., Gładysz O., Szentner K., Goliński P. (2014). Role of Glutathione in Abiotic Stress Tolerance. Oxid. Damage Plants Antioxid. Netw. Signal..

[11] Vivancos P.D., Wolff T., Markovic J., Pallardo F.V., Foyer C.H. (2010). A nuclear glutathione cycle within the cell cycle. Biochem. J..

[12] Diaz-Vivancos P., De Simone A., Kiddle G., Foyer C.H. (2015). Glutathione—Linking cell proliferation to oxidative stress. Free Radic. Biol. Med..

[13] Cai Y., Cao F., Cheng W., Zhang G., Wu F. (2011). Modulation of exogenous glutathione in phytochelatins and photosynthetic performance against cd stress in the two rice genotypes differing in Cd tolerance. Biol. Trace Elem. Res..

[14] Schnaubelt D., Schulz P., Hannah M.A., Yocgo R.E., Foyer C.H. (2013). A phenomics approach to the analysis of the influence of glutathione on leaf area and abiotic stress tolerance in *Arabidopsis thaliana*. Front. Plant Sci..

[15] Singla-Pareek S.L., Yadav S.K., Pareek A., Reddy M.K., Sopory S.K. (2006). Transgenic tobacco overexpressing glyoxalase pathway enzymes grow and set viable seeds in zinc-spiked soils. Plant Physiol..

[16] Iannelli M.A., Pietrini F., Fiore L., Petrilli L., Massacci A. (2002). Antioxidant response to cadmium in *Phragmites australis* plants. Plant Physiol. Biochem..

[17] Hossain M.A., Hasanuzzaman M., Fujita M. (2010). Up-regulation of antioxidant and glyoxalase systems by exogenous glycinebetaine and proline in mung bean confer tolerance to cadmium stress. Physiol. Mol. Biol. Plants.

[18] Hossain M.A., Piyatida P., da Silva J.A.T., Fujita M. (2012). Molecular mechanism of heavy metal toxicity and tolerance in plants: Central role of glutathione in detoxification of reactive oxygen species and methylglyoxal and in heavy metal chelation. J. Bot..

[19] Chen F., Wang F., Wu F.B., Mao W.H., Zhang G.P., Zhou M.X. (2010). Modulation of exogenous glutathione in antioxidant defense system against Cd stress in the two barley genotypes differing in Cd tolerance. Plant Physiol. Biochem..

[20] Mostofa M.G., Hossain M.A., Fujita M., Tran L.S.P. (2015). Physiological and biochemical mechanisms associated with trehalose-induced copper-stress tolerance in rice. Sci. Rep..

[21] Li M., Hao P.F., Cao F.B. (2017). Glutathione-induced alleviation of cadmium toxicity in *Zea mays*. Plant Physiol. Biochem..

[22] Lushchak V.I. (2011). Adaptive response to oxidative stress: Bacteria, fungi, plants and animals. Comp. Biochem. Phys. C.

[23] Shahid M., Pourrut B., Dumat C., Nadeem M., Aslam M., Pinelli E. (2014). Heavy-metal-induced reactive oxygen species: Phytotoxicity and physicochemical changes in plants. Rev. Environ. Contam. Toxicol..

[24] Shahid M.A., Balal R.M., Khan N., Zotarelli L., Liu G.D., Sarkhosh A., Fernandez-Zapata J.C., Nicolas J.J.M., Garcia-Sanchez F. (2019). Selenium impedes cadmium and arsenic toxicity in potato by modulating carbohydrate and nitrogen metabolism. Ecotoxicol. Environ. Saf..

[25] Sevcikova H., Maskova P., Tarkowska D., Masek T., Lipavska H. (2017). Carbohydrates and gibberellins relationship in potato tuberization. J. Plant Physiol..

[26] Shahjee H.M., Banerjee K., Ahmad F. (2002). Comparative analysis of naturally occurring L-amino acid osmolytes and their D-isomers on protection of *Escherichia coli* against environmental stresses. J. Biosci..

[27] Murakeozy E.P., Nagy Z., Duhaze C., Bouchereau A., Tuba Z. (2003). Seasonal changes in the levels of compatible osmolytes in three halophytic species of inland saline vegetation in Hungary. J. Plant Physiol..

[28] Sarraf N.S., Saboury A.A., Ranjbar B., Nemat-Gorgani M. (2005). Effect of some amino acids on the structure and activity of carbonic anhydrase. Asian J. Chem..

[29] Sun X.M., Zhang J.X., Zhang H.J., Ni Y.W., Zhang Q., Chen J.P., Guan Y.F. (2010). The responses of *Arabidopsis thaliana* to cadmium exposure explored via metabolite profiling. Chemosphere.

[30] Zhou Y., Wen Z.L., Zhang J.W., Chen X.J., Cui J.X., Xu W., Liu H.Y. (2017). Exogenous glutathione alleviates salt-induced oxidative stress in tomato seedlings by regulating glutathione metabolism, redox status, and the antioxidant system. Sci. Hortic. Amst..

[31] Pisoschi A.M., Pop A., Cimpeanu C., Predoi G. (2016). Antioxidant Capacity Determination in Plants and Plant-Derived Products: A Review. Oxid. Med. Cell. Longev..

[32] Michalak A. (2006). Phenolic compounds and their antioxidant activity in plants growing under heavy metal stress. Pol. J. Environ. Stud..

[33] Mianabadi M., Hoshani M., Salmanian S. (2015). Antimicrobial and Anti-oxidative Effects of Methanolic Extract of Dorema aucheri Boiss. J. Agric. Sci. Technol. Iran..

[34] Quartacci M.F., Argilla A., Baker A.J.M., Navari-Izzo F. (2006). Phytoextraction of metals from a multiply contaminated soil by Indian mustard. Chemosphere.

[35] Mohamed A.A., Castagna A., Ranieri A., Sanita di Toppi L. (2012). Cadmium tolerance in *Brassica juncea* roots and shoots is affected by antioxidant status and phytochelatin biosynthesis. Plant Physiol. Biochem..

[36] Navarro-Reig M., Jaumot J., Pina B., Moyano E., Galceran M.T., Tauler R. (2017). Metabolomic analysis of the effects of cadmium and copper treatment in *Oryza sativa* L. using untargeted liquid chromatography coupled to high resolution mass spectrometry and all-ion fragmentation. Metallomics.

[37] Deng W.K., Wang Y.B., Liu Z.X., Cheng H., Xue Y. (2014). HemI: A Toolkit for Illustrating Heatmaps. PLoS ONE.

