# Supplementary material for: Metabolome Analysis Revealed the Mechanism of Exogenous Glutathione to Alleviate Cadmium Stress in Maize (Zea mays L.) Seedlings
Source: Plants (Basel). 2021 Jan 6;10(1):105. doi: 10.3390/plants10010105 (PMC7825527; doi:10.3390/plants10010105)
Supplement: Supplementary file 1 [file plants-10-00105-s001.pdf]

Supplementary information

Metabolome Analysis Revealed the Mechanism of Exogenous Glutathione to Alleviate Cadmium Stress in Maize (*Zea mays* L.) Seedlings

Runfeng Wang[1, §], Kaina Lin[1, §], Huabin Chen[1], Zhenyu Qi[2], Bohan Liu[1, 3], Fangbin Cao[1, *], Hao Chen[1,*], Feibo Wu[1]

[1] Institute of Crop Science, College of Agriculture and Biotechnology, Zhejiang University, Yu-Hang-Tang Road No 866, Hangzhou 310058, China; 11516025@zju.edu.cn (R.W.), linkaina@zju.edu.cn (K.L.), 21816033@zju.edu.cn (H.C.), liubohan@zju.edu.cn (B.L.) and wufeibo@zju.edu.cn (F.W.)

[2] Experimental Station, Zhejiang University, Yu-Hang-Tang Road No 866, Hangzhou 310058, China; qizhenyu@zju.edu.cn (Z.Q.)

[3] Southern Regional Collaborative Innovation Center for Grain and Oil Crops in China, College of Agriculture, Hunan Agricultural University, Changsha 410128, China; liubohan@hunau.edu.cn (B.L.)

[*]Correspondence: caofangbin@zju.edu.cn (F.C.) and haochen@zju.edu.cn (H.C.).

[§] These authors contributed equally to this work.

Table S1. Different metabolites in the leaves and roots of maize seedlings under cadmium stress and exogenous GSH conditions.

Metabolites	FC (CdL)	FC (CdR)	FC (GSHL)	FC (GSHR)
Prunin degr. prod. 1	-2.37	-2.17	2.29	0.90
Sorbitol	2.38	-	-3.01	-
D-Altrose 1	0.33	0.54	-1.26	0.33
o-Hydroxyhippuric acid 2	-0.21	2.69	-1.02	0.84
Caffeic acid	0.86	-0.64	-0.76	2.27
Linoleic acid methyl ester	0.90	0.84	-0.74	1.99
D-(glycerol 1-phosphate)	1.74	0.22	-0.72	0.47
β-Alanine 2	0.74	-1.03	-0.66	-0.23
Aconitic Acid	0.31	3.69	-0.61	-3.30
Lactic acid	-0.27	1.81	-0.55	-0.39
Maltose	1.24	1.02	-0.55	0.05
Myo-inositol	2.37	1.19	-0.53	1.27
Lysine	1.67	0.40	-0.46	-0.12
Glucuronic acid 2	2.24	-1.66	-0.46	-0.93
Glucose-1-phosphate	0.51	1.02	-0.44	-0.54
O-Phosphorylethanolamine	0.26	-1.67	-0.44	-0.48
Shikimic acid	0.24	1.32	-0.43	-0.26
2-Deoxy-D-glucose 2	4.71	0.15	-0.42	-0.83
3,6-Anhydro-D-galactose 1	1.05	0.96	-0.42	0.19
Threonic acid	0.21	1.00	-0.41	-2.89
Flavanone 1	-0.19	1.49	-0.40	1.66
Glutathione 1	0.60	-1.10	-0.30	2.34
Tyrosine 1	1.40	-2.20	-0.28	-0.36
Fructose 2,6-biphosphate degr. prod	0.34	0.63	-0.27	1.71
2-Aminophenol 2	-0.14	2.48	-0.26	-1.09
Turanose 1	1.31	-0.03	-0.24	-0.22
Stearic acid	0.12	-0.09	-0.23	2.23
2-Deoxyerythritol	-1.39	-	-0.21	-
Aspartic acid 2	1.21	-1.38	-0.16	1.63
Linoleic acid	0.74	0.27	-0.15	3.42
Gentiobiose 1	-0.09	-0.75	-0.15	1.27
Aspartic acid 1	3.38	-2.54	-0.13	1.56
Conduritol b epoxide 2	0.17	0.41	-0.03	-1.62
Palmitic acid	0.90	-0.77	-0.02	1.65
2-Methylfumarate	0.13	-1.00	-0.01	0.58
4-Hydroxycinnamic acid	0.41	-0.11	0.02	-1.18
Ferulic acid	0.66	1.68	0.05	0.60
Prostaglandin A2 1	1.44	-	0.06	-
3-Methylamino-1,2-propanediol 2	-0.25	1.10	0.07	-2.71
Arbutin	1.71	0.29	0.08	-0.01
Piceatannol 1	2.32	-0.01	0.08	-0.86

4-Nitrophenol	-2.12	1.57	0.09	1.83
Citric acid	1.34	0.59	0.10	-0.15
Gluconic lactone 3	-0.60	-1.29	0.12	0.15
Methyl-beta-D-galactopyranoside	1.06	0.05	0.12	-0.37
Glutamic acid	0.48	-1.31	0.16	0.19
Gluconic acid 1	0.25	1.05	0.17	-
Quinic acid	0.14	1.02	0.19	-0.19
Glycine 2	-1.85	-1.56	0.21	-0.70
N-Acetyl-L-phenylalanine 1	1.22	-0.14	0.25	-0.38
Urocanic acid 1	0.82	-1.48	0.26	0.94
4-Pyridoxic acid	1.04	-0.25	0.28	-2.58
α-Ketoglutaric acid	-0.78	1.75	0.28	-0.61
Palatinitol 1	1.07	0.12	0.29	0.12
Tartaric acid	2.28	0.14	0.33	-1.07
4-Aminobutyric acid 1	-1.12	-0.66	0.38	-0.43
Fructose 1	0.41	1.34	0.39	0.86
Methyl trans-cinnamate	-0.23	-1.12	0.39	0.78
2-Deoxy-D-galactose 2	1.27	0.24	0.50	-0.09
Guanine 1	0.73	0.47	0.52	1.54
Ascorbate	-0.89	-0.24	0.54	-2.22
Succinic acid	-1.24	0.22	0.65	-0.39
Glucose-6-phosphate 1	1.13	0.22	0.66	1.72
L-Glutamic acid	-1.21	-	0.73	0.34
6-Deoxy-D-glucose 1	-1.05	-	0.80	-
Oxamic acid	-0.81	-1.91	0.81	0.25
Adenosine	-1.61	0.17	0.92	0.31
Glucoheptonic acid 3	-0.43	0.11	0.94	2.26
D-Glucoheptose 1	-1.74	0.23	1.16	1.75
Sophorose 2	-1.00	-0.23	1.20	0.26
3-Phosphoglycerate	-0.61	-	1.35	0.06
L-Malic acid	-1.49	1.14	1.35	-0.52
Maleic acid	-1.06	0.16	1.46	0.09
Allo-inositol	-1.04	-	1.64	-
Sucrose	-2.23	0.20	1.84	-0.28
Gentisic acid	-	-3.51	-	2.13
Thymidine 1	-0.61	-2.21	-	0.70
Lyxonic acid, 1,4-lactone	-	1.25	-	-1.57
Phenyl beta-D-glucopyranoside	0.97	1.43	-	-0.42

FC: Fold changes (Cd vs control; GSH vs Cd) in $\log_2 N$, $\log_2 N \geq 1$ are increased, $0 < |\log_2 N| < 1$ are unchanged and $\log_2 N \leq -1$ are decreased. CdL, Cd treatment vs control in leaves; CdR, Cd treatment vs control in roots; GSHL, exogenous GSH and Cd treatment vs Cd treatment in leaves; and GSHR, exogenous GSH and Cd treatment vs Cd treatment in roots. Cd treatment (5 μM $CdCl_2$), exogenous GSH (30 μM GSH).
